# On the Treatment of Scabies and Some Other Common Skin Affections in Soldiers

**Published:** 1918-01

**Authors:** 


***On the Treatment of Scabies and Some Other Common Skin Affections in Soldiers.*** By H. G. Adamson, Hon. Consultant for skin diseases in Military Hospitals in London. Abs. from the *Lancet*, Feb. 10, 1917.

Pediculosis, scabies, and impetigo contagiosa, the author finds, are, next to the venereal diseases, the most common skin affections in the army. Others that might naturally be expected are strangely lacking. Psoriasis and ring-worm are also troublesome.

*Scabies.* — When a man complains of itching, especially at night, it is well to think of scabies. Burrows between the fingers, about the wrist, upon the buttocks, and upon the penis, causing red raised ridges, are characteristic. On the fingers and wrist the burrow appears as a narrow wavy line, from 1/16 to 1/4 of an inch long, and often black from dirt. Generally near the end of the burrow there is a clear vesicle the size of a pinhead, over which the burrow passes. If examined microscopically, the acarus looks like a minute, smooth, flat pebble, imbedded in the skin. It can be removed on the end of a darning-needle. Examination of the acarus microscopically in a little liquor potassae, confirms the diagnosis. An inflamed burrow on the penis is an unmistakable sign.

Scabies is often complicated, chiefly with impetigo contagiosa and eczema. Then the burrow and the acarus are more difficult to detect. When complicated with impetigo, the hands and feet particulary are the seat of vesicles and bullae with clear or turbid contents, together with crusts, erosions, and inflamed burrows; and there may be crusted infiltrated nodules (ecthyma) on the buttocks, and often crusts and erosions on other parts, and sometimes a small furuncle.

When eczema complicates scabies there are red, rough, eczematoid areas — sometimes dry and sometimes weeping — on the hands, arms, thighs, and trunk, masking often the burrows of the original complaint. The eczema may sometimes result from a too prolonged use of sulphur applications or from scratching.

As to transmission, the author remarks that scabies is usually a contact disease, but that it may also be conveyed by such things as playing cards, dominoes, and utensils.

*Treatment.* — For hardly any curable disease have so many remedies been suggested. The sulphur treatment, which, although dating from the seventeenth century, is coming into special favor to-day, is effective, but dangerous if used for too long a time.

The author recommends the use of sulphur ointment after a bath with soft soap; but only to a strictly limited extent, so as to avoid eczema. His method is as follows: (1) A hot bath of half-hour duration for three consecutive days. The whole body, as well as the affected parts, should be scrubbed with soft soap and a fresh brush. (2) After each bath the patient himself rubs in the ointment and puts on an old sleeping suit, gloves, and socks. He remains in bed until the time for the bath the next day.

The three days' treatment requires:

| | |
|---|---|
| Saponis mollis. . . . . . . . . . . . . | 24 oz. (700 gm.) |
| Ung. sulphuris. . . . . . . . . . . . | 24 oz. (700 gm.) |

This treatment should not under any circumstances be continued after the third day, but if thought necessary, the following lotion may be used night and morning:

| | |
|---|---|
| Lic. picis carbonis. . . . . . . . . . . | 1 dr. (4 cc.) |
| Aq. . . . . . . . . . . . . . . . . . . . | 20 oz. (600 cc.) |

If these methods do not result in cure, the trouble is probably not scabies but either papular urticaria or chronic eczema. For the former, a lotion of ordinary vinegar may suffice. For the eczema, a free application of zinc ointment without baths is advisable:

| | |
|---|---|
| Zinci oxidi. . . . . . . . . . . . . . | 1 oz. (30 gm.) |
| Pulv. amyli. . . . . . . . . . . . . . | 1 oz. (30 gm.) |
| Vaselin. . . . . . . . . . . . . . . . . . | 10 oz. (300 gm.) |

Disinfection of clothing has proved unnecessary, providing that outer garments are properly aired and brushed, and undergarments washed.

*Impetigo contagiosa.* — Its usual situation is the face, beard region, scalp, and fingers. It may be acquired at the barber's or from a public towel. It generally appears in the beard region as isolated crusts which spread quickly to other parts of the face and head. It is easily confused with sycosis or eczema. It may be identified by the nature of its crusts which are amber-colored, of the " stuck on " variety, resulting from rapidly drying serum blisters, although the original blisters are rarely seen. When the crusts are removed by forceps, a weeping surface is disclosed. A phlyctenular whitlow may appear at the root of the finger or thumb nails, and crusted patches upon the hands or forearms.

The eruption is of streptococcic origin. If properly treated it may be cured in a few days : if not, a later staphylococcic infection may occur in the form of sycosis.

*Treatment.* — The patient should be made to mop off the crusts with a wad of wool and hot water, and to continue to mop for half an hour or until every crust is removed. A lotion of 1 to 6000 hydrarg. perchlor. is then daubed on for a few minutes and a diluted white precipitate ointment applied (hydrarg. ammon. chlor. 10 gr. (0.6 gm.), vaseline 1 oz. (30 gm.)). The baths and applications of the ointment should be continued several times daily until the cure is complete. Great care, however, must be taken to remove all scabs before applying the treatment. If the staphylococcic complication of sycosis occurs, it may be checked by a timely use of staphylococcal vaccines, otherwise an incurable condition may result.

*Ringworm.* — This affection is less common and appears in several varieties, most commonly on the groin and extremities. For any of its forms the author recommends a formula prescribed by Dr. Arthur Whitfield :

| | |
|---|---|
| Acid. benzoici. . . . . . . . . . . . . . | 15 gr. (1 gm.) |
| Acid. salicylici. . . . . . . . . . . . . . | 15 gr. (1 gm.) |
| Ol. coca-nucis. . . . . . . . . . . . . . | 3 dr. (12 gm.) |
| Vaselin. . . . . . . . . . . . . . . . . | 1 oz. (30 gm.) |

For especially obstinate cases the author recommends treatment as follows :

Thoroughly mop the affected parts with tinctura iodi; then rub on argent. nitratis 10 gr. (0.6 gr.), spirit. ætheris nitrosi 1 oz. (30 gm.). Repeat this treatment if necessary, and apply the benzoic acid ointment between whiles.

*Psoriasis.* — Internal treatments are useless. The eruption may be reduced by rubbing in the following ointment twice daily with a piece flannel :

| | |
|---|---|
| Hydrarg. ammon. chlor. . . . . . . . | 1 dr. (5 gm.) |
| Liq. picis carbonis. . . . . . . . . . . | 1/2 oz. (15 cc.) |
| Ung. paraffin. B. P. . . . . . . . . . | 6 oz. (180 gm.) |

If this ointment fails, recourse may be had to ung. chrysarobini B. P., but care should be exercised to avoid face, hands, and scalp.

The patient must be kept in bed, and the treatment stopped if chrysarobin erythema appears.

